# Crystal structures of glutamyl-tRNA synthetase from *Elizabethkingia anopheles* and *E. meningosepticum*

**DOI:** 10.1107/S2053230X22007555

**Published:** 2022-07-28

**Authors:** Lauryn Brooks, Sandhya Subramanian, David M. Dranow, Stephen J. Mayclin, Peter J. Myler, Oluwatoyin A. Asojo

**Affiliations:** a Department of Chemistry and Biochemistry, Hampton University, Hampton, VA 23668, USA; b Center for Global Infectious Disease Research, Seattle Children’s Research Institute, 307 Westlake Avenue North Suite 500, Seattle, WA 98109, USA; c Seattle Structural Genomics Center for Infectious Disease (SSGCID), Seattle, Washington, USA; d UCB-Bainbridge, Bainbridge Island, WA 98110, USA; e Departments of Pediatrics, Global Health, and Biomedical Informatics and Medical Education, University of Washington, Seattle, Washington, USA; Osaka University, Japan

**Keywords:** glutamyl-tRNA synthetases, undergraduate education and training, Seattle Structural Genomics Center for Infectious Disease, infectious diseases, *Elizabethkingia meningosepticum*, *Elizabethkingia anopheles*, emerging infectious diseases

## Abstract

*Elizabethkingia* bacteria are globally emerging pathogens that cause opportun­istic and nosocomial infections, with up to 40% mortality among the immuno­compromised. *Elizabethkingia* species are in the pipeline of organisms for high-throughput structural analysis at the Seattle Structural Genomics Center for Infectious Disease (SSGCID). These efforts include the structure–function analysis of potential therapeutic targets. Glutamyl-tRNA synthetase (GluRS) is essential for tRNA aminoacylation and is under investigation as a bacterial drug target. The SSGCID produced, crystallized and determined high-resolution structures of GluRS from *E. meningosepticum* (*Em*GluRS) and *E. anopheles* (*Ea*GluRS). *Em*GluRS was co-crystallized with glutamate, while *Ea*GluRS is an apo structure. *Em*GluRS shares ∼97% sequence identity with *Ea*GluRS but less than 39% sequence identity with any other structure in the Protein Data Bank. *Em*GluRS and *Ea*GluRS have the prototypical bacterial GluRS topology. *Em*GluRS and *Ea*GluRS have similar binding sites and tertiary structures to other bacterial GluRSs that are promising drug targets. These structural similarities can be exploited for drug discovery.

## Abstract 2

*Elizabethkingia* bacteria cause opportunistic infections in neonates, the elderly and the immunocompromised with mortality rates of up to 40%. The high-resolution structures of glutamyl-tRNA synthetase (GluRS) from *E. meningosepticum* and *E. anopheles* reveal similarities to bacterial GluRSs that can be exploited to accelerate rational drug discovery for these globally important emerging infectious Gram-negative bacteria.

## 1. Introduction

*Elizabethkingia* are Gram-negative, obligate aerobic bacilli that were first described in 1959 by Elizabeth O. King. *Elizabethkingia* bacteria were previously classified as *Chryseobacterium* or *Flavobacterium*, so there is some variability in their nomenclature in the literature (Kim *et al.*, 2005 ▸). *Elizabethkingia* are widely found in the environment, in soils, rivers and insect vectors, and have even been isolated from condensation water on the International Space Station (Li *et al.*, 2003 ▸; Weon *et al.*, 2008 ▸; Bevivino *et al.*, 2014 ▸; Dziuban *et al.*, 2018 ▸). While *Elizabethkingia* species rarely cause disease in the healthy, they are now globally recognized as causing opportunistic infections in neonates, the elderly and the immunocompromised, with mortality rates ranging from 18% to 40% (Dziuban *et al.*, 2018 ▸; Lin *et al.*, 2019 ▸). *Elizabethkingia* infections usually lead to meningitis, sepsis, bacteremia, lower respiratory tract infection, pneumonia, pneumothorax, endocarditis, cellulitis, endophthalmitis, keratitis, wound infection after bone fractures, and urinary-tract infections (Singh *et al.*, 2020 ▸; Lin *et al.*, 2019 ▸; Jean *et al.*, 2020 ▸).

*E. anopheles* was initially isolated from *Anopheles* mosquitoes and causes respiratory-tract illnesses in adults and neonatal meningitis in premature infants, with a notable outbreak in 2016 in Wisconsin (Figueroa Castro *et al.*, 2017 ▸). Before 2016, it was believed that *E. meningosepticum* (formerly *F. meningosepticum* or *C. meningosepticum*) was the predominant human pathogen of the genus. A study of past *Elizabethkingia* outbreaks revealed that most nosocomial infections were caused by *E. anopheles* (Figueroa Castro *et al.*, 2017 ▸). Routine phenotypic and biochemical tests often fail to distinguish between *E. anopheles* and *E. meningosepticum*. Additionally, the misidentification of *E. anopheles* is mainly attributed to the absence of updated MALDI–TOF reference-spectrum databases; thus, genome sequencing is recommended for correct identification at the species and sublineage level (Nielsen *et al.*, 2018 ▸). Antibiotics such as piperacillin–tazobactam and cotrimoxazole have proven efficacy against other *Elizabethkingia* species, while *E. anopheles* and *E. meningosepticum* cause multidrug-resistant infections (Patro *et al.*, 2021 ▸; Baruah *et al.*, 2020 ▸).

The Seattle Structural Genomics Center for Infectious Disease (SSGCID) includes *E. anopheles* and *E. meningosepticum* among the priorities for rational drug discovery. These efforts include the identification and structure–function characterization of proteins, such as glutamyl-tRNA synthet­ase (GluRS), as possible targets for drug repurposing and identification. GluRS catalyzes tRNA aminoacylation: the binding of glutamate to tRNA. GluRS and other aminoacyl-tRNA synthetases are crucial for bacterial survival and are promising targets for drug discovery for infectious diseases (Kwon *et al.*, 2019 ▸; Lee *et al.*, 2018 ▸; Moen *et al.*, 2017 ▸). Here, the production, crystallization and high-resolution structures of GluRS from *E. meningosepticum* (*Em*GluRS) and *E. anopheles* (*Ea*GluRS) are reported.

## 2. Materials and methods

### 2.1. Macromolecule production

Cloning, expression and purification followed standard protocols as described previously (Bryan *et al.*, 2011 ▸; Choi *et al.*, 2011 ▸; Serbzhinskiy *et al.*, 2015 ▸). The full-length GluRS genes from *E. anopheles* (*Ea*GluRS; UniProt A0A077E909) and *E. meningosepticum* (*Em*GluRS; UniProt R9CN54) encoding amino acids 1–503 were PCR-amplified from gDNA using the primers given in Table 1 ▸. Each gene was cloned using ligation-independent cloning (LIC) encoding a noncleavable hexahistidine tag (MAHHHHHH-ORF; Aslanidis & de Jong, 1990 ▸; Choi *et al.*, 2011 ▸). Plasmid DNA was transformed into chemically competent *Escherichia coli* BL21(DE3)R3 Rosetta cells. The plasmid containing His-*Ea*GluRS or His-*Em*GluRS was tested for expression, and 2 l of culture were grown using auto-induction medium (Studier, 2005 ▸) in a LEX Bioreactor (Epiphyte Three) as described previously (Serbzhinskiy *et al.*, 2015 ▸). The expression clones ElanA.01348.a.B1.41090 and ElmeA.01348.a.B1.GE41608 are available at https://www.ssgcid.org/available-materials/expression-clones/.

His-*Ea*GluRS and His-*Em*GluRS were purified in a two-step protocol consisting of an immobilized metal (Ni^2+^) affinity chromatography (IMAC) step and size-exclusion chromatography (SEC). All chromatography runs were performed on an ÄKTApurifier 10 (GE Healthcare) using automated IMAC and SEC programs (Bryan *et al.*, 2011 ▸). Thawed bacterial pellets (∼25 g) were lysed by sonication in 200 ml buffer consisting of 25 m*M* HEPES pH 7.0, 500 m*M* NaCl, 5% glycerol, 0.5% CHAPS, 30 m*M* imidazole, 10 m*M* MgCl_2_, 1 m*M* TCEP, 250 µg ml^−1^ AEBSF, 0.025% sodium azide. After sonication, the crude lysate was clarified with 20 ml (25 units µl^−1^) benzonase and incubated while mixing at room temperature for 45 min. The lysate was clarified by centrifugation at 10 000 rev min^−1^ for 1 h using a Sorvall centrifuge (Thermo Scientific). The clarified supernatant was then passed over an Ni–NTA HisTrap FF 5 ml column (GE Healthcare) which was pre-equilibrated with loading buffer composed of 25 m*M* HEPES pH 7.0, 500 m*M* NaCl, 5% glycerol, 30 m*M* imidazole, 1 m*M* TCEP, 0.025% sodium azide. The column was washed with 20 column volumes (CV) of loading buffer and was eluted with loading buffer plus 250 m*M* imidazole in a linear gradient over 7 CV. Peak fractions were pooled and concentrated to 5 ml. A SEC column (Superdex 75, GE Healthcare) was equilibrated with a running buffer consisting of 25 m*M* HEPES pH 7.0, 500 m*M* NaCl, 5% glycerol, 2 m*M* DTT, 0.025% sodium azide. The peak fractions were collected and analyzed using SDS–PAGE for the protein of interest. Both proteins eluted as a single large peak at a molecular mass of ∼50 kDa, suggesting a monomeric enzyme. The peak fractions were pooled and concentrated to 36.5 mg ml^−1^ (His-*Ea*GluRS) and 16.23 mg ml^−1^ (His-*Em*GluRS) using an Amicon purification system (Millipore). Aliquots of 200 µl were flash-frozen in liquid nitrogen and stored at −80°C until use.

### 2.2. Crystallization

Purified His-*Ea*GluRS and His-*Em*GluRS were screened for crystallization in 96-well plates against JBScreen JCSG++ HTS (Jena Bioscience) and MCSG1 (Molecular Dimensions) crystal screens. Equal volumes of protein solution (0.4 µl) and precipitant solution were set up at 290 K against reservoir (80 µl) in sitting-drop vapor-diffusion format. The crystals were flash-cooled by harvesting them and plunging them directly into liquid nitrogen with or without additional cryoprotection depending on whether the precipitant solution had been supplemented with 20% ethylene glycol (Table 2 ▸).

### 2.3. Data collection and processing

Data were collected at 100 K on beamline 21-ID-F at the Advanced Photon Source, Argonne National Laboratory (Table 3 ▸). Data were integrated with *XDS* and reduced with *XSCALE* (Kabsch, 2010 ▸). Raw X-ray diffraction images for 6b1z are available at the Integrated Resource for Repro­ducibility in Macromolecular Crystallography at https://www.proteindiffraction.org (https://doi.org/10.18430/M36B1Z).

### 2.4. Structure solution and refinement

The structure of *Em*GluRS was determined by molecular replacement with *Phaser* (McCoy *et al.*, 2007 ▸) from the *CCP*4 suite of programs (Collaborative Computational Project, 1994 ▸; Krissinel *et al.*, 2004 ▸; Winn *et al.*, 2011 ▸) using domains of PDB entries 4gr1 (Janes & Schulz, 1990 ▸), 2ja2 (G. P. Bourenkov, N. Strizhov, L. A. Shkolnaya, M. Bruning, H. D. Bartunik, unpublished work) and 2qmz (Y. Fu, L. Buryanovskyy & Z. Zhang, unpublished work) as search models. The structure of *Ea*GluRS was solved using *MR-Rosetta* (Terwilliger *et al.*, 2012 ▸) with PDB entry 2ja2 as the search model. Both structures were refined with *phenix.refine* (Adams *et al.*, 2011 ▸) followed by manual structure rebuilding using *Coot* (Emsley & Cowtan, 2004 ▸; Emsley *et al.*, 2010 ▸). The quality of each structure was checked using *MolProbity* (Williams *et al.*, 2018 ▸). A representative quality of electron density is illustrated in Supplementary Fig. S1. Data-reduction and refinement statistics are shown in Table 4 ▸. Coordinates and structure factors have been deposited with the Worldwide PDB (wwPDB) as entries 6b1z and 6brl.

## 3. Results and discussion

**Figure 1:**
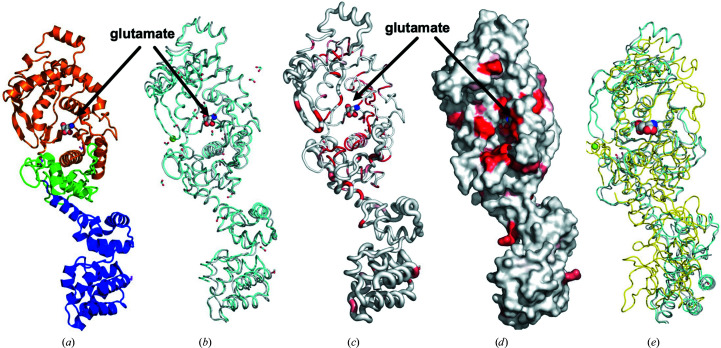
Structures of *Em*GluRS and *Ea*GluRS. (*a*) The *Em*GluRS monomer has a Rossmann fold (orange), a zinc-binding domain (green) and an anticodon-binding domain (blue). The Rossmann fold and zinc-binding domain make up the N-terminal tRNA synthetase binding domain that binds the glutamate (spheres). (*b*) Superposed structures of *Em*GluRS (gray) and *Ea*GluRS (cyan). The Mg^2+^ ion in *Ea*GluRS is shown as a green sphere, the glutamate molecule is shown as spheres (C atoms in gray, O atoms in red and N atoms in blue) and formate and ethylene glycol from crystallization are shown as sticks. (*c*) Ribbon diagram calculated by *ENDScript*. The circumference of the ribbon (sausage) represents the relative structural conservation compared with other GluRS structures (these structures are indicated in Supplementary Fig. S2). Thinner ribbons represent more highly conserved regions, while thicker ribbons represent less conserved regions. (*d*) Solvent-accessible surface area of *Em*GluRS colored by sequence conservation, with red indicating identical residues. (*e*) Superposed structures of *Pa*GluRS (PDB entry 5tgt, yellow), *Em*GluRS (gray) and *Ea*GluRS (cyan). The sequence alignment of *Pa*GluRS is shown in Fig. 3 ▸.

The structures of *Elizabethkingia* GluRSs reported here share ∼97% sequence identity. *Em*GluRS and *Ea*GluRS are monomeric enzymes that assemble with a prototypical GluRS topology with an N-terminal tRNA synthetase class I (E and Q) catalytic domain and a C-terminal anticodon-binding domain (Fig. 1 ▸). The tRNA synthetase class I (E and Q) catalytic domain consists of a Rossmann-fold domain (Aravind *et al.*, 2002 ▸) containing a glutamate-binding domain and a zinc-binding domain (Fig. 1 ▸). There is a glutamate molecule in the glutamate-binding domain of *Em*GluRS and a divalent ion (Mg^2+^) in the zinc-binding domain of *Ea*GluRS (Fig. 1 ▸). The *Em*GluRS and *Ea*GluRS structures are very similar and have a root-mean-squared difference of ∼1.3 Å for the alignment of all main-chain C^α^ atoms.

**Figure 2:**
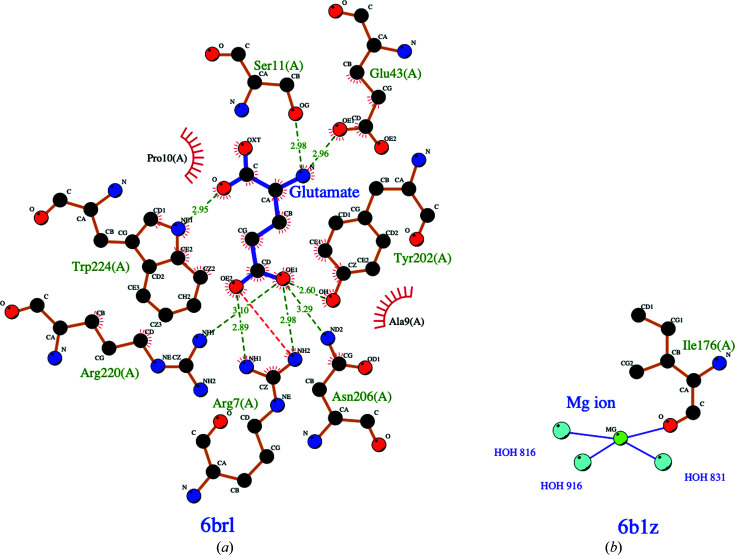
*LigPlot* representations of (*a*) glutamate binding and (*b*) Mg^2+^ ion binding in *Em*GluRS and *Ea*GluRS, respectively.

*ENDScript* (Gouet *et al.*, 2003 ▸; Robert & Gouet, 2014 ▸) analyses revealed that despite having <40% sequence similarity, *Em*GluRS and *Ea*GluRS share significant secondary-structural similarity with other bacterial GluRSs and other aminoacyl-tRNA synthetases, including some that have shown promise as drug targets (Supplementary Fig. S2). The N-terminal tRNA synthetase binding domains of all of these proteins have a sizeable accessible glutamate-binding site that is evident in the surface plot (Fig. 1 ▸ *d*). The glutamate-binding region is highly conserved, as indicated by the red color in the ribbon and surface *ENDScript* plots (Figs. 1 ▸ *c* and 1 ▸ *d*). *PDBeFold* analysis (http://www.ebi.ac.uk/msd-srv/ssm/; Krissinel & Henrick, 2004 ▸) using default thresholds of 70% validated the *ENDScript* analysis, showing well conserved bacterial GluRSs (Supplementary Table S1). The amino-acid residues involved in glutamate binding in *Em*GluRS and in cation binding in *Ea*GluRS are indicated in the *LigPlot* diagrams (Laskowski & Swindells, 2011 ▸; Wallace *et al.*, 1995 ▸; Fig. 2 ▸).

**Figure 3:**
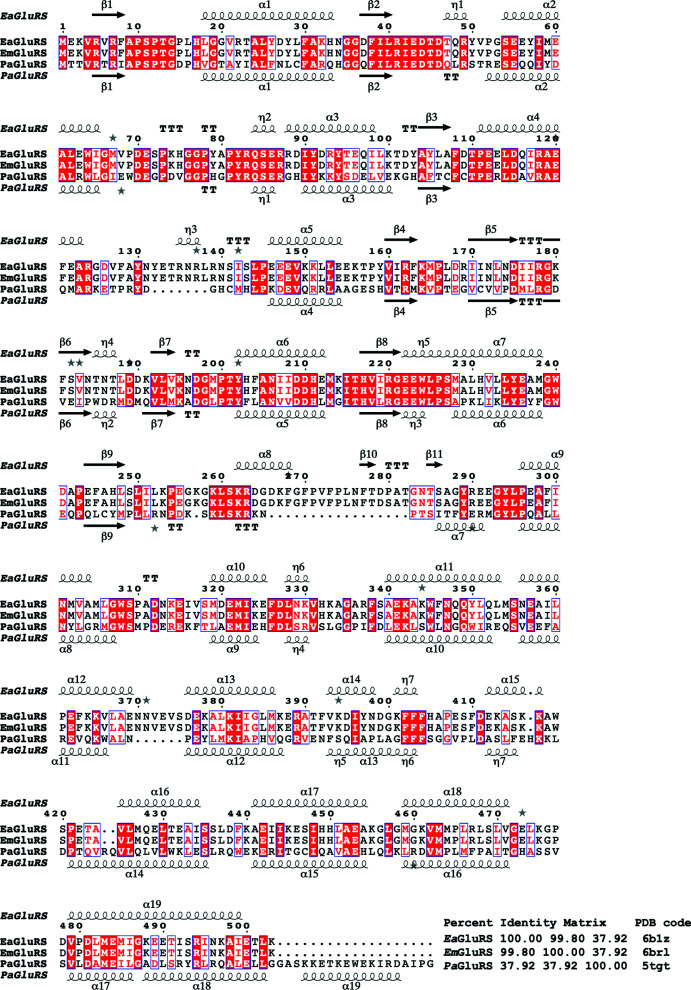
Structural and primary-sequence alignment of *Ea*GluRS, *Em*GluRS and *Pa*GluRS. The secondary-structure elements are as follows: α-helices are shown as large coils, 3_10_-helices are shown as small coils labeled η, β-strands are shown as arrows labeled β and β-turns are labeled TT. Identical residues are shown on a red background, with conserved residues in red and conserved regions in blue boxes. This figure was generated using *ESPript* (Gouet *et al.*, 1999 ▸, 2003 ▸).

It has previously been shown that bacterial GluRSs are promising targets for drug discovery (Kwon *et al.*, 2019 ▸; Lee *et al.*, 2018 ▸; Moen *et al.*, 2017 ▸). Intriguingly, the glutamate-binding cavity has been probed to develop promising inhibitors for *Pseudomonas aeruginosa* GluRS (*Pa*GluRS; Hu *et al.*, 2015 ▸). *Pa*GluRS has a similar structural topology to *Ea*GluRS and *Em*GluRS (Fig. 3 ▸ *a*). The residues that bind glutamate in the binding cavity are identical (Fig. 3 ▸ *b*) despite the low sequence identity (37.9%) between *Pa*GluRS and *Ea*GluRS and *Em*GluRS. Additionally, residues in proximity to the glutamate-binding cavity are also well conserved. These residues are also conserved in other bacterial GluRSs (Supplementary Fig. S2). These observations suggest that the lessons learned from rational inhibitory design for *Pa*GluRS and other bacterial GluRSs can also be applied to *Ea*GluRS and *Em*GluRS.

## 4. Conclusion

We report the production, crystallization and structures of GluRS from *E. meningosepticum* (*Em*GluRS) and *E. anopheles* (*Ea*GluRS). *Em*GluRS and *Ea*GluRS are prototypical bacterial GluRSs with well conserved glutamate-binding cavities. Their structural similarity to the well studied *P. aeruginosa* GluRS and the lessons learned from other bacterial GluRSs can be exploited to develop potential inhibitors for these emerging infectious agents.

## Supplementary Material

PDB reference: glutamyl-tRNA synthetase from *E. anopheles*, 6b1z

PDB reference: glutamyl-tRNA synthetase from *E. meningosepticum*, complex with glutamate, 6brl

Supplementary Table and Figures. DOI: 10.1107/S2053230X22007555/nw5116sup1.pdf

Raw diffraction data.: https://doi.org/10.18430/M36B1Z

## Figures and Tables

**Table 1:** Macromolecule-production information

	*Ea*GluRS	*Em*GluRS
Source organism	*Elizabethkingia anopheles* NUHP1	*Elizabethkingia meningosepticum* CCUG 26117
DNA source	Dr Yang Liang (Nanyang Technological University, Singapore)	ATCC 13253
Forward primer	5′-CTCACCACCACCACCACCATATGGAAAAAGTACGGGTACGTTTTG-3′	
Reverse primer	5′-ATCCTATCTTACTCACTTATTTTAAAGTTTCAATTGCTTTATTAATTC-3′	
Expression vector	pBG1861	BG1861
Expression host	*E. coli* BL21(DE3)R3 Rosetta cells	*E. coli* BL21(DE3)R3 Rosetta cells
Complete amino-acid sequence of the construct produced	MAHHHHHHMEKVRVRFAPSPTGPLHLGGVRTALYDYLFAKHNGGDFILRIEDTDTQRYVPGSEEYIMEALEWIGMVPDESPKHGGPYAPYRQSERRDIYDRYTEQILKTDYAYLAFDTPEELDQIRAEFEARGDVFAYNYETRNRLRNSISLPEEEVKKLLEEKTPYVIRFKMPLDRIINLNDIIRGKFSVNTNTLDDKVLVKNDGMPTYHFANIIDDHEMKITHVIRGEEWLPSMALHVLLYEAMGWDAPEFAHLSLILKPEGKGKLSKRDGDKFGFPVFPLNFTDPATGNTSAGYREEGYLPEAFINMVAMLGWSPADNKEIVSMDEMIKEFDLNKVHKAGARFSAEKAKWFNQQYLQLMSNEAILPEFKKVLAENNVEVSDEKALKIIGLMKERATFVKDIYNDGKFFFHAPESFDEKASKKAWSPETAVLMQELTEAISSLDFKAEIIKESIHHLAEAKGLGMGKVMMPLRLSLVGELKGPDVPDLMEMIGKEETISRINKAIETLK	MAHHHHHHMEKVRVRFAPSPTGPLHLGGVRTALYDYLFAKHNGGDFILRIEDTDTQRYVPGSEEYIMEALEWIGMIPDESPKHGGPYAPYRQSERRAIYDKYTEQILKTDYAYLAFDTPEELDQIRAEYEAKGDVFAYNYETRHRLRNSISLPEDEVKKLLDEKTPYVIRFKMPLDRIINLNDIIRGKFSVNTNTLDDKVLVKNDGMPTYHFANIIDDHEMKITHVIRGEEWLPSMALHVLLYEAMEWNAPEFAHLSLILKPEGKGKLSKRDGDKFGFPVFPLNFTDPATGNTSAGYREEGYLPEAFINMVAMLGWSPADNKEIISMDEMIKEFDLHKVHKAGARFSAEKAKWFNQQYLQMMSNEAILPEFKTILSNNSIEISDEKALRIIGLMKERATFIKDIYNDGKFFFHAPESYDEKAAKKAWSPETAALMQEVNNAITTVDFKADTIKESLHHLTEEKGLGMGKVMMPLRLSLVGELKGPDVPELMEIIGKEESVSRITKAIETLK

**Table 2:** Crystallization

	His-*Ea*GluRS	His-*Em*GluRS
Method	Sitting-drop vapor diffusion	Sitting-drop vapor diffusion
Plate type	96-well, Compact 300, Rigaku	96-well, Compact 300, Rigaku
Temperature (K)	290	290
Protein concentration (mg ml^−1^)	18.25	16.23
Buffer composition of protein solution	25 m*M* HEPES pH 7.0, 500 m*M* NaCl, 5% glycerol, 2 m*M* DTT, 0.025% sodium azide	
Composition of reservoir solution	JBScreen JCSG++ HTS A5: 0.2 *M* magnesium formate, 20%(*w*/*v*) PEG 3350	MCSG1 E10: 200 m*M* ammonium tartarate dibasic, 20%(*w*/*v*) PEG 3350
Volume and ratio of drop	0.4 µl protein plus 0.4 µl reservoir (1:1)	0.4 µl protein plus 0.4 µl reservoir (1:1)
Volume of reservoir (µl)	80	80
Cryoprotectant	20% ethylene glycol	None

**Table 3:** Data collection and processing Values in parentheses are for the outer shell.

	*Ea*GluRS	*Em*GluRS
Ligand	—	Glutamic acid
Diffraction source	Beamline 21-ID-F, APS	Beamline 21-ID-F, APS
Wavelength (Å)	0.97872	0.97872
Temperature (K)	100	100
Detector	Rayonix MX-300 CCD	Rayonix MX-300 CCD
Crystal-to-detector distance (mm)	200	240
Rotation range per image (°)	1	1
Total rotation range (°)	150	150
Space group	*P*2_1_2_1_2_1_	*P*2_1_2_1_2_1_
*a*, *b*, *c* (Å)	47.17, 99.78, 132.59	43.26, 111.89, 130.17
Mosaicity (°)	0.198	0.183
Resolution range (Å)	50–1.60 (1.64–1.60)	50–2.00 (2.05–2.00)
Total No. of reflections	503995 (37374)	265391 (19568)
No. of unique reflections	83273 (6107)	43563 (3169)
Completeness (%)	99.7 (100.0)	99.8 (99.9)
Multiplicity	6.05 (6.12)	6.09 (6.17)
〈*I*/σ(*I*)〉	26.5 (3.5)	17.7 (3.2)
*R*_r.i.m._	0.039 (0.50)	0.069 (0.62)
Overall *B* factor from Wilson plot (Å^2^)	20.1	31.1

**Table 4:** Structure solution and refinement Values in parentheses are for the outer shell.

	*Ea*GluRS	*Em*GluRS
Ligand	—	Glutamic acid
Resolution range (Å)	50–1.60 (1.64–1.60)	50–2.00 (2.05–2.00)
Completeness (%)	97.2	99.8 (99.9)
σ Cutoff	0.00σ(*F*)	1.35σ(*F*)
No. of reflections, working set	81099 (5241)	43551 (2922)
No. of reflections, test set	1941 (125)	1997 (136)
Final *R*_cryst_	0.178 (0.211)	0.168 (0.213)
Final *R*_free_	0.211 (0.261)	0.214 (0.255)
Cruickshank DPI	0.094	0.411
No. of non-H atoms		
Protein	3838	3947
Ion	1	—
Ligand	76	12
Solvent	579	404
Total	4494	4373
R.m.s. deviations		
Bond lengths (Å)	0.006	0.012
Angles (°)	0.76	1.09
Average *B* factors (Å^2^)		
Protein	31.6	37.1
Ion	21.8	—
Ligand	55.0	51.8
Water	40.7	44.6
Ramachandran plot		
Most favored (%)	98	99
Allowed (%)	2	1
