# Supplementary material for: Crystal structures of glutamyl-tRNA synthetase from Elizabethkingia anopheles and E. meningosepticum
Source: Acta Crystallogr F Struct Biol Commun. 2022 Jul 28;78(Pt 8):306–12. doi: 10.1107/S2053230X22007555 (PMC9350836; doi:10.1107/S2053230X22007555)
Supplement: Supplementary file 1 [file f-78-00306-sup1.pdf]

Acta Cryst F

STRUCTURAL BIOLOGY COMMUNICATIONS

Volume 78 (2022)

Supporting information for article:

Crystal structures of glutamyl-tRNA synthetase from *Elizabethkingia anopheles* and *E. meningosepticum*

Lauryn Brooks, Sandhya Subramanian, David M. Dranow, Stephen J. Mayclin, Peter J. Myler and Oluwatoyin A. Asojo

*

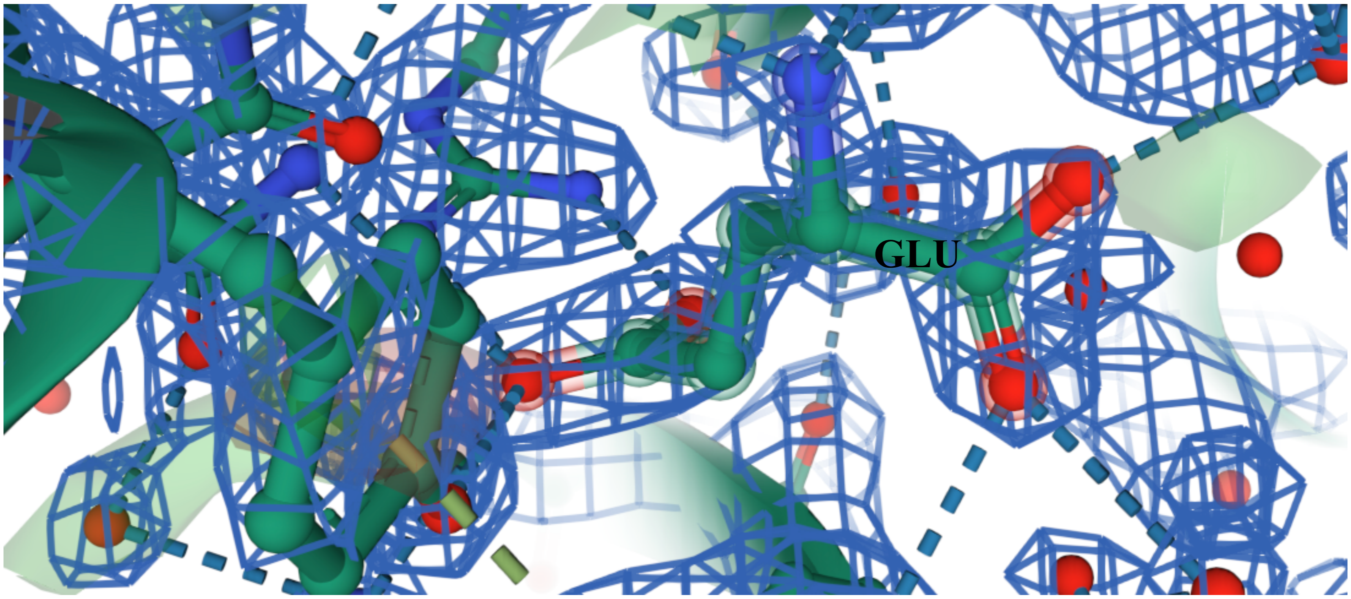

Figure S1 Quality of final 2Fo -Fc electron density map around glutamate in 6BRL contoured at 1.5sigma cutoff

References

Gouet, P., Robert, X. & Courcelle, E. (2003). *Nucleic Acids Res* **31**, 3320-3323.
Krissinel, E. & Henrick, K. (2004). *Acta crystallographica. Section D, Biological crystallography* **60**, 2256-2268.
Robert, X. & Gouet, P. (2014). *Nucleic Acids Res* **42**, W320-324.

Table S.1 Results from PDBeFold P (http://www.ebi.ac.uk/msd-srv/ssm/) analysis (Krissinel & Henrick, 2004)

PDBe Fold v2.59. (src3) 14 Apr 2014 result file.

RESULT SUMMARY

##	Q-score	P-score	Z-score	RMSD	Nalgn	Nsse	Ngaps	Seq-%	Nmd	Nres-Q	Nsse-Q	Nres-T	Nsse-T	Query	Target
1	0.8372	55.43	22.5	1.169	490	29	5	0.9673	0	495	31	503	33	PDB 6b1z:A	PDB 6brl:A
2	0.5865	25.7	16.52	1.955	440	25	12	0.3841	0	495	31	468	29	PDB 6b1z:A	PDB 2cv1:B
3	0.5829	24.7	16.22	1.998	442	27	15	0.3914	0	495	31	469	30	PDB 6b1z:A	PDB 1n78:B
4	0.5814	26.29	16.67	1.962	439	27	13	0.385	0	495	31	469	31	PDB 6b1z:A	PDB 2cv2:B
5	0.5728	25.58	16.49	1.905	432	25	14	0.3912	0	495	31	469	29	PDB 6b1z:A	PDB 2dxi:B
6	0.569	25.28	16.9	1.934	432	25	15	0.3958	0	495	31	468	30	PDB 6b1z:A	PDB 2cv0:B
7	0.5473	23.75	15.96	2.031	430	27	17	0.3767	0	495	31	468	31	PDB 6b1z:A	PDB 1g59:C
8	0.5445	23.81	16.45	2.002	427	25	16	0.3864	0	495	31	468	29	PDB 6b1z:A	PDB 1n77:B
9	0.5313	20.55	15.07	1.997	429	23	21	0.338	0	495	31	485	30	PDB 6b1z:A	PDB 2cfo:A
10	0.5305	19.05	14.2	2.078	435	23	18	0.3517	0	495	31	487	30	PDB 6b1z:A	PDB 2cfo:B
11	0.5241	19.47	14.68	2.221	434	25	15	0.3825	0	495	31	469	29	PDB 6b1z:A	PDB 2dxi:A
12	0.5226	19.17	14.59	2.244	435	25	14	0.3839	0	495	31	469	29	PDB 6b1z:A	PDB 2cv2:A
13	0.5218	18.96	14.35	2.362	443	27	16	0.3792	0	495	31	469	29	PDB 6b1z:A	PDB 1n78:A
14	0.5208	19.18	14.59	2.262	435	25	14	0.3839	0	495	31	468	29	PDB 6b1z:A	PDB 1n77:A
15	0.5206	20.96	14.93	2.321	440	25	15	0.3841	0	495	31	470	28	PDB 6b1z:A	PDB 2cv0:A
16	0.5126	18.18	14.28	2.298	435	25	14	0.3839	0	495	31	470	29	PDB 6b1z:A	PDB 2cv1:A
17	0.5115	17.8	14.17	2.202	427	27	16	0.3817	0	495	31	468	30	PDB 6b1z:A	PDB 1g59:A
18	0.4705	14.57	13.76	2.351	427	23	17	0.3396	0	495	31	485	32	PDB 6b1z:A	PDB 3pny:B
19	0.4683	17.64	13.94	2.373	420	27	17	0.3476	0	495	31	468	30	PDB 6b1z:A	PDB 2o5r:A
20	0.4654	14.29	13.59	2.297	421	23	19	0.3444	0	495	31	485	31	PDB 6b1z:A	PDB 3pnv:B
21	0.4631	13.1	12.6	2.636	436	27	17	0.367	0	495	31	468	31	PDB 6b1z:A	PDB 1gln:A
22	0.4522	12.04	12.83	2.430	424	23	20	0.3349	0	495	31	485	30	PDB 6b1z:A	PDB 2ja2:A
23	0.4471	12.01	13.11	2.446	421	24	23	0.342	0	495	31	481	31	PDB 6b1z:A	PDB 3pnv:A
24	0.4353	14.72	13.2	2.438	417	25	20	0.3645	0	495	31	486	30	PDB 6b1z:A	PDB 4gri:B
25	0.4322	16.79	14.09	2.313	400	23	15	0.405	0	495	31	469	30	PDB 6b1z:A	PDB 2cuz:A
26	0.4298	10.79	12.69	2.523	418	23	22	0.3349	0	495	31	481	31	PDB 6b1z:A	PDB 3pny:A
27	0.4288	16.7	14.26	2.298	397	23	15	0.4081	0	495	31	468	30	PDB 6b1z:A	PDB 1n75:A
28	0.4228	14.12	13.53	2.362	403	25	19	0.3747	0	495	31	479	30	PDB 6b1z:A	PDB 4gri:A
29	0.4176	14.84	13.64	2.326	394	24	16	0.401	0	495	31	469	30	PDB 6b1z:A	PDB 1j09:A
30	0.3354	7.152	10.14	3.131	399	25	21	0.3434	0	495	31	459	28	PDB 6b1z:A	PDB 7k86:A
31	0.3124	8.429	10.82	2.891	372	24	28	0.3468	0	495	31	464	29	PDB 6b1z:A	PDB 7k86:B

Figure S.2 Results from ENDScript alignment (Gouet et al., 2003, Robert & Gouet, 2014)

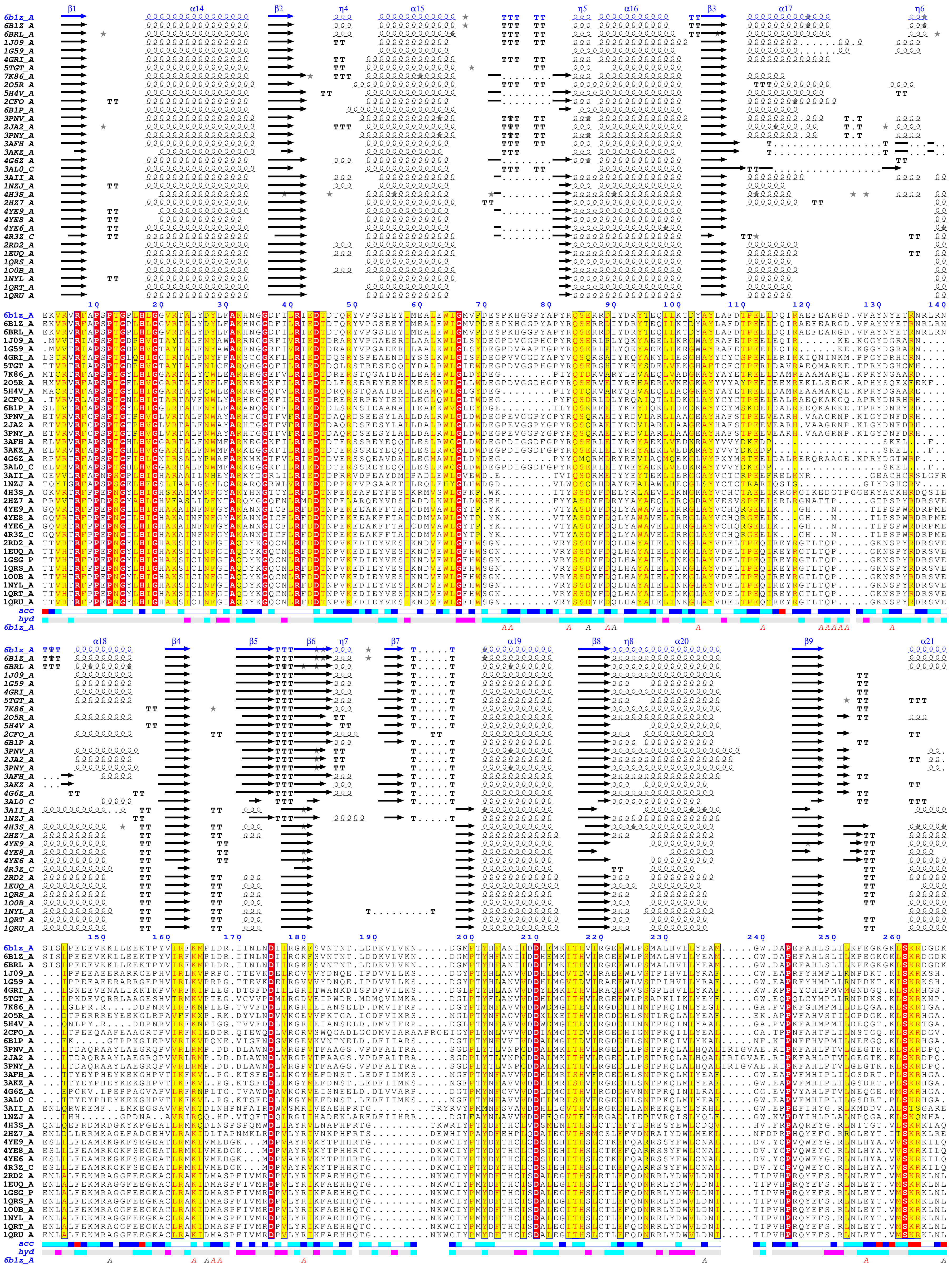

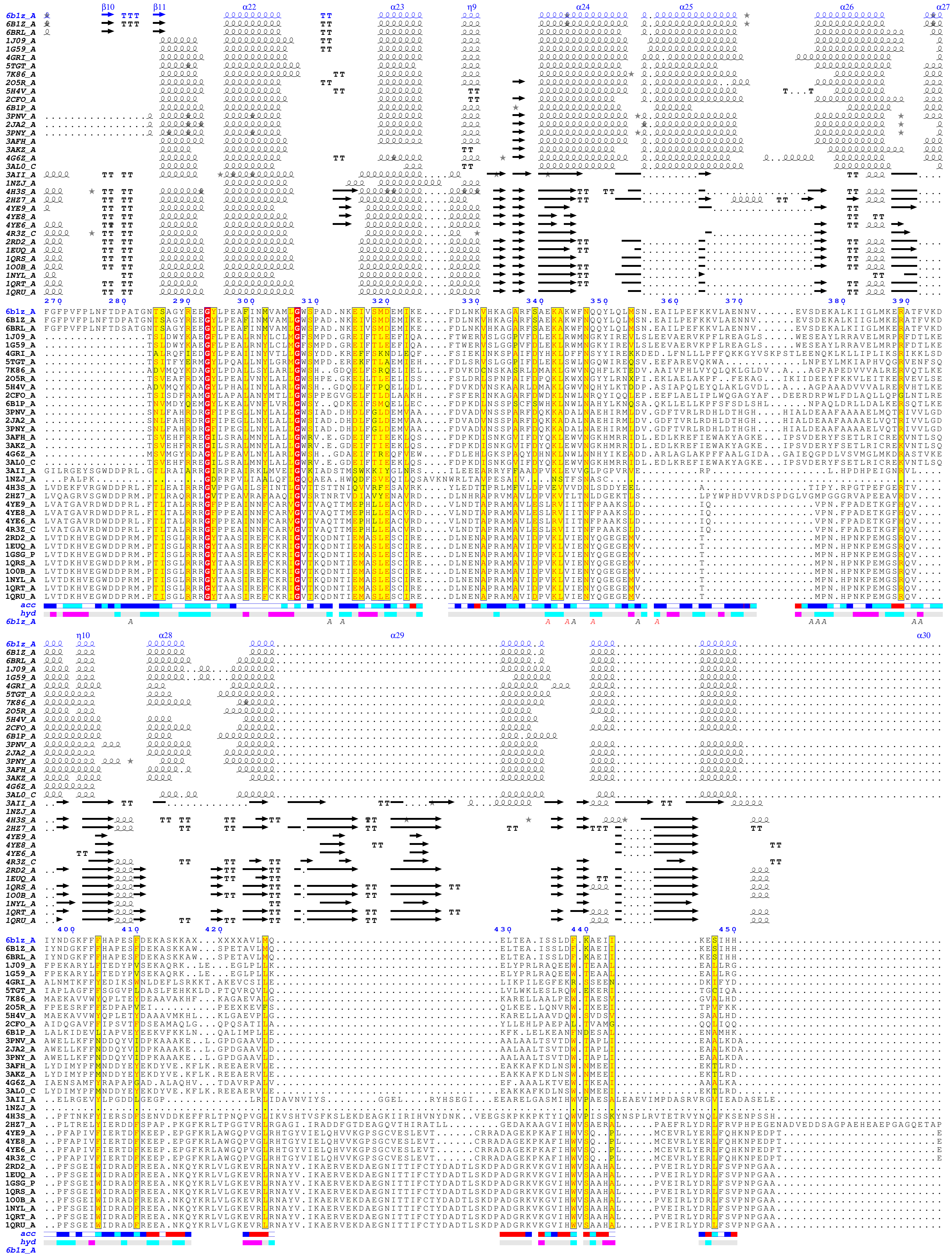
β10 β11 α22 α23 η9 α24 α25 α26 α27
6b1z_A
6B1Z_A
6BRL_A
1J09_A
1G59_A
4GRI_A
5TGT_A
7K86_A
2O5R_A
5H4V_A
2CFO_A
6B1P_A
3PNV_A
2JA2_A
3PNY_A
3AFH_A
3AKZ_A
4G6Z_A
3AL0_C
3AII_A
1NZJ_A
4H3S_A
2HZ7_A
4YE9_A
4YE8_A
4YE6_A
4R3Z_C
2RD2_A
1EUQ_A
1GSG_P
1QRS_A
1O0B_A
1NYL_A
1QRT_A
1QRU_A
270 280 290 300 310 320 330 340 350 360 370 380 390
acc
hyd
η10 α28 α29 α30
400 410 420 430 440 450

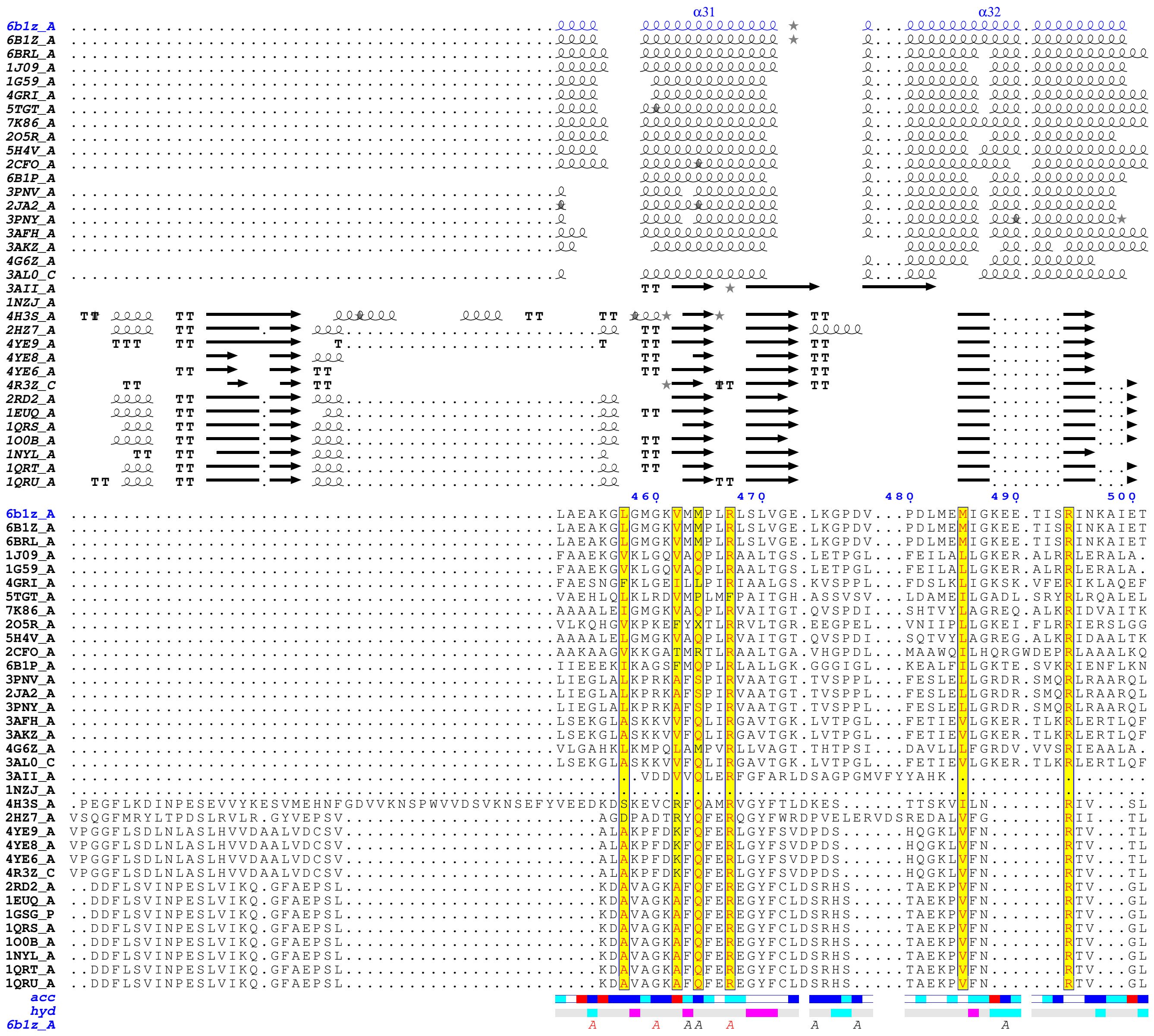
α31
α32
460
470
480
490
500
acc
hyd
